# Pharmacotherapy as an Augmentation to Bariatric Surgery for Obesity

**DOI:** 10.1007/s40265-024-02029-0

**Published:** 2024-07-06

**Authors:** Luděk Horváth, Miloš Mráz, Edward B. Jude, Martin Haluzík

**Affiliations:** 1 Diabetes Centre, Institute for Clinical and Experimental Medicine, Prague, Czech Republic; 2 Tameside and Glossop Integrated Care NHS Foundation Trust, Ashton-under-Lyne and University of Manchester, Manchester, United Kingdom

## Abstract

A global obesity pandemic is one of the most significant health threats worldwide owing to its close association with numerous comorbidities such as type 2 diabetes mellitus, arterial hypertension, dyslipidemia, heart failure, cancer and many others. Obesity and its comorbidities lead to a higher rate of cardiovascular complications, heart failure and increased cardiovascular and overall mortality. Bariatric surgery is at present the most potent therapy for obesity, inducing a significant weight loss in the majority of patients. In the long-term, a substantial proportion of patients after bariatric surgery experience a gradual weight regain that may, in some, reach up to a presurgical body weight. As a result, anti-obesity pharmacotherapy may be needed in some patients after bariatric surgery to prevent the weight regain or to further potentiate weight loss. This article provides an overview of the use of anti-obesity medications as an augmentation to bariatric surgery for obesity. Despite relatively limited published data, it can be concluded that anti-obesity medication can serve as an effective adjunct therapy to bariatric surgery to help boost post-bariatric weight loss or prevent weight regain.

## Key Points

**Table:** 

Modern treatment of obesity is based on a multicomponent approach that includes lifestyle changes, anti-obesity medication and, in some cases, bariatric surgery.
Bariatric surgery is the most effective treatment of obesity but there is still a risk of weight regain in some patients after surgery.
Anti-obesity medication provides an effective adjunct therapy modality to attenuate weight regain and to augment post-bariatric weight loss.

## Introduction

**Fig. 1:**
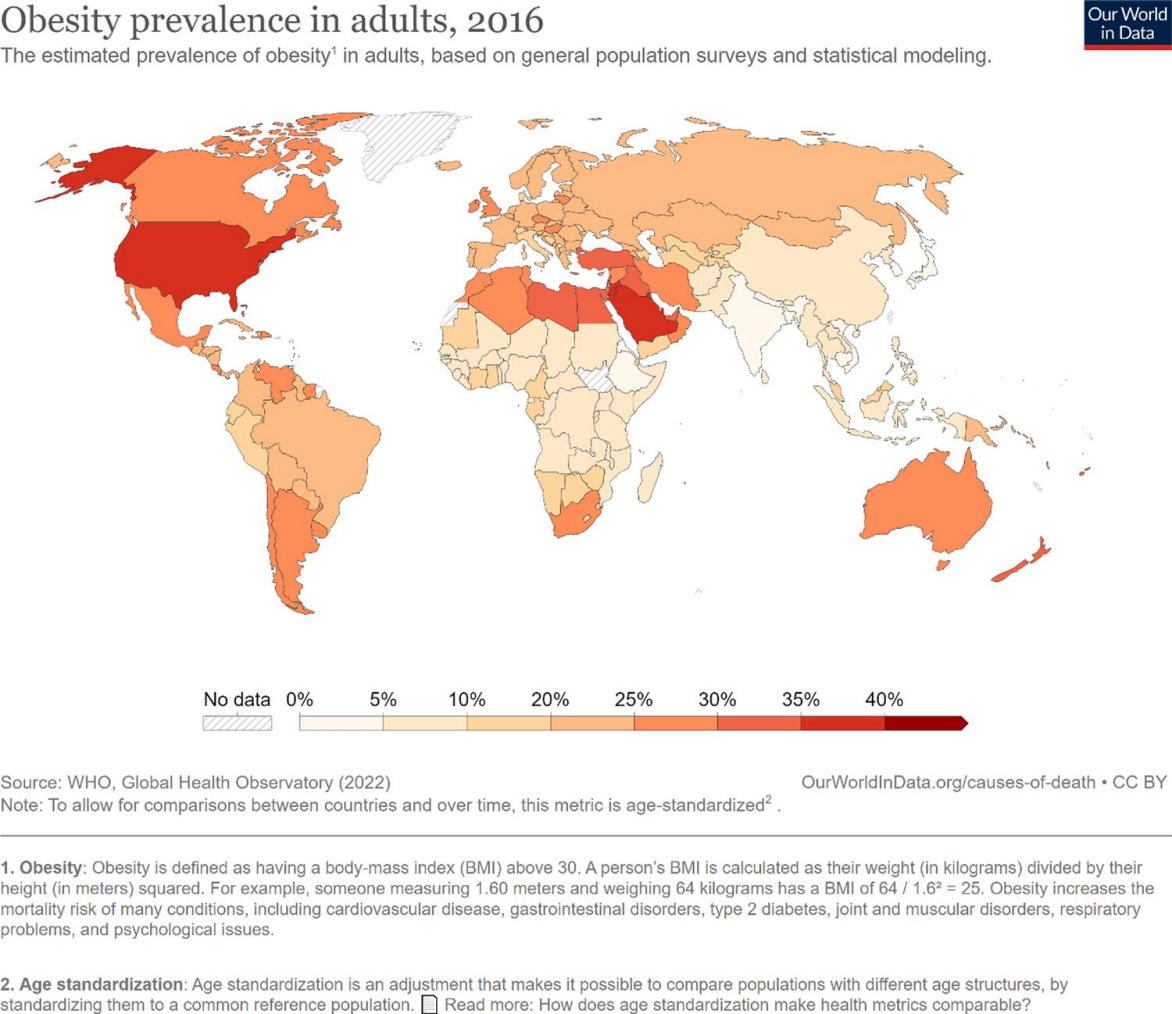
Global obesity prevalence in 2016 [155]

In the modern, postindustrial age, obesity stands as one of the most globally prevalent diseases of affluence. For decades, obesity has been spreading both geographically and demographically, straining both the healthcare and socioeconomic systems of afflicted countries [1] (Fig. 1).

The rate of spread of obesity has been especially accelerated by the coronavirus disease 2019 (COVID-19) pandemic and by the resulting lockdown, with a further rise in obesity that is most evident in the pediatric population [2].

While obesity is highly prevalent in most parts of the world, the situation is the most pressing in the Americas, Middle East, parts of Europe, southeast Asia, and the Pacific Islands [3]

Epidemiological World Health Organization (WHO) data have shown that in 2016 approximately 67.9% (95% confidence interval [CI] 64.5–71.1) of the adult population in the United States (US) had a body mass index (BMI) in the overweight or obesity range [4], while 36.2% of adults (95% CI 32.3–40.1) have reached BMI values characteristic of obesity [3]. These values are closely followed by Europe, where 58.7% (95% CI 56.9–60.5) of the adult population has been diagnosed with overweight or obesity, and 23.3% (95% CI 21.7–24.9) had a BMI in the obese category (Fig. 1, Table 1)

**Table 1:** Classification of obesity

Category	BMI (kg/m^2^)	Waist-to-hip ratio according to WHO—Women	Waist-to-hip ratio according to WHO—Men
Underweight	<18.5	X	X
Normal	18.5–24.9	X	X
Overweight	25–29.9	X	X
Class 1 obesity	30–34.9	>0.85	>0.90
Class 2 obesity	35–39.9	X	X
Class 3 obesity	≥40	X	X

*BMI* body mass index, *WHO* World Health Organization

The obesity pandemic is of great concern, as obesity has been linked to the development of numerous comorbidities, including hypertension [5], dyslipidemia [6], type 2 diabetes mellitus [7, 8], atrial fibrillation [9], non-alcoholic fatty liver disease (NAFLD) [10], sleep apnea syndrome [11], cancer [12] and a wide spectrum of other conditions [13–15]. The incidence of obesity-associated comorbidities generally increases proportionally with BMI, with at least some of these comorbidities being partially or completely reversible by significant weight loss [5, 16].

While the treatment of obesity has traditionally been built on lifestyle and dietary changes, with the addition of bariatric surgery in relatively rare cases, the modern approach is based on a step-up principle consisting of lifestyle modifications, anti-obesity medication and bariatric surgery if needed [17].

When comparing the effectiveness of weight loss regimens, multiple parameters can be used, with the most common being pure weight loss (kg), BMI loss (kg/m^2^), total weight loss (TWL, %) and excess weight loss (EWL, %). Excess body weight (kg) is calculated as actual weight minus expected weight if BMI was 25 kg/m^2^ [18].

The aim of this review was to compare the efficacy and feasibility of the use of anti-obesity medication in patients after bariatric surgery. Additionally, this article will provide a general introduction to bariatric surgery and pharmacodynamics of anti-obesity medication.

## Bariatric Surgery

Bariatric surgery is recognized as the most effective treatment for obesity [19] and type 2 diabetes mellitus, leading to long-term diabetes remission in a high percentage of patients [20], with one study reporting diabetes remission in 74% of patients undergoing Roux-en-Y gastric bypass (RYGB) 1 year after surgery [21], with other trials reporting similar outcomes [22, 23].

According to the 2022 joint statement of the American Society for Metabolic and Bariatric Surgery (ASMBS) and International Federation for the Surgery of Obesity and Metabolic Disorders (IFSO), the criteria for bariatric surgery are either BMI ≥ 35 kg/m^2^ or BMI ≥ 30 kg/m^2^ in the presence of metabolic disease associated with obesity. The 2022 statement also proposes a lower threshold of BMI ≥ 27.5 kg/m^2^ for the Asian population, in response to the proposed lower obesity threshold of ≥ 25 kg/m^2^ in this population [24]. The 2023 National Institute for Health and Care Excellence (NICE) draft guidelines on obesity (CG189) are stricter, with the criteria for assessment being BMI ≥ 40 kg/m^2^, BMI ≥ 35 kg/m^2^ with a health condition that could be improved by weight loss, or BMI ≥ 30 kg/m^2^ with a recent-onset (≤ 10 years) type 2 diabetes mellitus. People of Asian, Chinese, other Asian, Middle Eastern, Black African or African-Caribbean ethnicity should be considered for an assessment at a reduced BMI (− 2.5 kg/m^2^) in all categories [25].

**Fig. 2:**
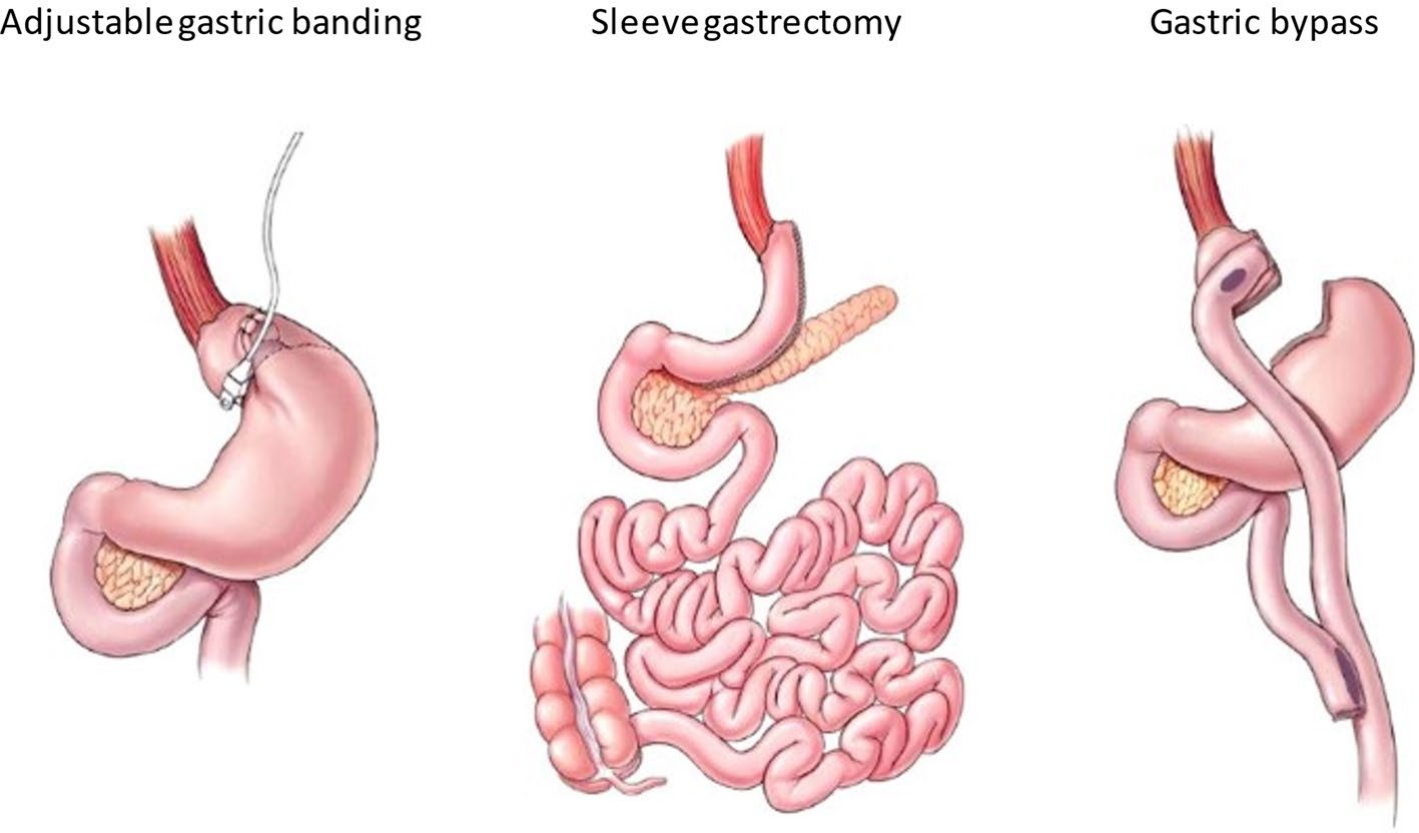
Selected bariatric surgery procedures

Bariatric surgery can be divided into three groups (malabsorptive, restrictive and combined), according to the expected general mechanism behind the weight loss. Malabsorptive surgeries work by excluding specific parts of the small intestine from the digestive process, while restrictive surgery, as the name implies, restricts the maximum amount of food one can consume in a given time period by decreasing the size of the stomach. Combined surgery includes both restrictive and malabsorptive mechanisms [26] (Fig. 2).

In spite of the high efficacy of bariatric surgery, post-bariatric weight regain is a common occurrence in the years after the operation. The rate of weight regain itself is difficult to quantify, as the definition of weight regain is not universally established. While some studies define weight regain as weight gain of 10 kg in addition to nadir weight [27–29], others use different metrics, such as a regain of at least 25% of maximum weight loss [30, 31].

Weight regain after bariatric surgery can be an indication for surgical revision, in the form of either corrective surgery (in the case of defective operation), or surgical expansion and/or conversion of the original operation. Examples of surgical expansions include adjustment of the gastric band [32], increasing the length of the non-alimentary limb in gastric bypass [33], or the conversion of sleeve gastrectomy (SG) to gastric bypass [34]

### Sleeve Gastrectomy

SG is the most common bariatric surgery type worldwide, accounting for 70% of bariatric surgeries in 2015 in the US [35]. SG, which started out as the first part of biliopancreatic diversion with duodenal switch, has become a stand-alone operation after the validation of its efficacy. It is based on the resection of a large part of the gastric fundus, reducing the gastric volume by 80–90%, with the residual gastric volume being around 170–190 mL [36, 37].

SG has gained popularity because of its low morbidity and mortality rate, significant weight loss, and the ease of conversion to other, more extensive surgeries in the case of insufficient efficacy and reversibility [38, 39]. While the original proposed mechanism behind this operation was pure restriction, it has become clear that the underlying mechanisms are more complex and include endocrine and neural mechanisms [26, 40].

According to a retrospective study (*n* = 13,900), the weight loss effect of SG at 1, 3 and 5 years was 27.98 kg (95% CI 28.27–27.63), 22.06 kg (95% CI 22.48–21.65) and 19.69 kg (95% CI 20.44–18.95), respectively [41]. The same study has shown the TWL at 1, 3 and 5 years was 22.98% (95% CI 23.19–22.76), 18.03% (95% CI 18.35–17.71) and 15.99% (95% CI 16.58–15.40), while BMI loss was 10.07 (95% CI 10.17– 9.97), 7.95 (95% CI 8.10–7.80) and 7.06 (95% CI 7.33–6.79) at 1, 3 and 5 years, respectively.

### Gastric Bypass

Gastric bypass as a group is the second most common bariatric surgery and the most common combined bariatric operation, with its most frequent variation, RYGB, and in 2015 encompassed 24% of bariatric surgeries in the US [35]. Before the introduction of the SG, the prevalence of RYGB was higher, with 45% of bariatric surgeries in the US being RYGB in 2010 [35]. Other gastric bypasses include single anastomosis sleeve ileal (SASI) bypass, single anastomosis duodeno-ileostomy (SADI) [42] and biliopancreatic diversion [37].

#### Roux-en-Y Gastric Bypass

RYGB involves the formation of a gastric pouch from the stomach, creation of an excluded biliopancreatic limb, anastomosis of the gastric pouch to the distal part of the jejunum via a gastrojejunostomy, and end-to-side anastomosis of the biliopancreatic limb to the alimentary limb [43, 44]. The length of the non-alimentary biliopancreatic limb can range from 60 to 200 cm [45], with the most common length being 60 cm [44].

According to a retrospective study (*n* = 17,258), the weight loss effect of RYGB at 1, 3 and 5 years, was 35.04 kg (95% CI 35.27–34.80), 31.20 kg (95% CI 31.53–30.88) and 27.34 kg (95% CI 27.73–26.96), respectively [41]. The same study has shown the TWL was 28.35% (95% CI 28.53–28.18), 24.83% (95% CI 25.06–24.60), and 21.74% (95% CI 22.02–21.45) at 1, 3 and 5 years, respectively, while the BMI loss was 12.81 (95% CI 12.90–12.72), 11.28 (95% CI 11.39–11.17), and 9.89 (95% CI 10.03–9.75), respectively.

While the longer-term (≥ 5 years) effectiveness of RYGB is higher than that of SG (62.58% EWL vs. 53.25%) [46], patients are at higher risk of surgical complications [47], dumping syndrome [48] and nutritional deficiencies [48], requiring lifelong vitamin and mineral supplementation.

#### Single Anastomosis Sleeve Ileal Bypass

SASI, also known as single anastomosis stomach ileal bypass with sleeve gastrectomy (SASI-S), is a combined bariatric surgery, consisting of SG with creation of an omega loop instead of a Roux-en-Y loop [49]. The omega loop is created by anastomosing the ileum to the stomach, with the gastroileal limb being approximately 300 cm in length [50]. The weight loss is achieved by both the effect of SG and accelerated contact of the ileum with the ingested food, leading to early anorexigenic signaling. Benefits of the surgery include preservation of the passage of nutrients throughout the entire intestine and the possibility of bypass reversal through closure of the anastomosis by a simple staple [51].

A pair-matched, retrospective study of patients after either SASI or SG (*n* = 58:58) reported the 1-year weight loss effect of SASI as a reduction of pre-bariatric weight of 131.3 kg ± 46.7 to post-bariatric weight of 81.7 kg ± 19.5. In comparison, preoperative weight in the SG group was 135.8 ± 63.9, with postoperative weight being 90.5 ± 12.5 (postoperative values, *p* = 0.004) [52]

The long-term weight loss effectiveness of SASI at the 1-, 2- and 4-year periods has been reported to be an EWL of 93.7%, 110.5%, and 93.3%, with TWL of 39.3%, 42.2%, and 41.2%, respectively, according to a retrospective monocentric study (*n* = 366). According to that study, no patients had a nutritional deficiency (hypovitaminosis, hypoalbuminemia) that did not respond to supplementation or higher protein uptake [53].

#### Single Anastomosis Duodeno-Ileostomy

SADI, also known as single anastomosis duodeno-ileostomy with sleeve gastrectomy (SADI-S) is a modification of biliopancreatic diversion with duodenal switch, consisting of an SG and a duodeno-ileostomy, created by connecting the stomach and proximal duodenum to the ileum with separation of the proximal and distal duodenum. This creates a blind-end biliopancreatic limb and a common channel, by which the biliopancreatic limb and the duodenum connect to the large intestine [54]. The length of the common channel can range from approximately 200–450 cm, with the most common length being 250 cm to prevent any heightened chance of nutritional deficiency [55, 56].

According to a prospective study (*n* = 31), the weight loss effect of SADI at 1 year was 43.5 kg (interquartile range [IQR] 16.0 kg), with an EWL of 72.6% (IQR 30.3%) [57]

Both SASI and SADI present a clear option for surgically treating weight loss failure or weight regain after SG by relatively simple expansion of the initial surgery [58, 59], albeit with an additional risk of surgical and anesthesiologic complications.

### Laparoscopic Adjustable Gastric Banding

Laparoscopic adjustable gastric banding (LAGB) is a minimally invasive laparoscopic bariatric surgery, consisting of implantation of an adjustable band in the proximal part of the stomach. The surgical technique itself involves insertion of a tube around the upper diameter of the stomach, it´s locking, suturing of the port to the aponeurosis and, after a specified time period, filling of the tube with fluid via the port, resulting in the creation of an upper gastric pouch proximally to the band [60]. However, LAGB has fallen from grace due to relatively low effectiveness, coupled with a high rate of complications and a frequent need for reoperation, and in 2014 it was required in only 1% of bariatric surgeries compared with 44% in 2018 [61].

A prospective randomized study (*n* = 80, 1:1) has reported a median 1-year weight after LAGB of 14 kg (IQR 5–38), while the weight loss after SG was 26 kg (IQR 0–46; *p* < 0.0001); the weight loss after 3 years was 17 kg (IQR 0–40) for LAGB and 29.5 kg (IQR 1–48) for SG (*p* < 0.0001) [62].

A meta-analysis has reported the long-term (5–10 years) effect of LAGB was an EWL of 47.94% (95% CI 45.31–50.57; *p* < 0.001), with a BMI reduction of 11.09 (95% CI 12.43–9.74; *p* < 0.001). When extended to a ≥10-year period, the EWL was 47.43% (95% CI 37.46–57.40; *p* < 0.001) and the BMI loss was 9.69 (95% CI 11.32–8.06; *p* < 0.001) [46].

## Pharmacological Treatment of Obesity

### Lorcaserin

While lorcaserin has been clinically used for the pharmacological treatment of obesity, it has been recalled by the US FDA due to safety concerns about increased occurrence of cancer. This article will therefore not discuss the use of lorcaserin as an anti-obesity medication [63].

### Phentermine/Topiramate

Phentermine is a sympathomimetic compound with structural and functional similarities to amphetamines [64, 65]. The primary mechanism of action is thought to be release of catecholamine neurotransmitters (predominantly noradrenaline) in the central nervous system (CNS), resulting mainly in appetite suppression, and possibly metabolism activation, via increased thermogenesis and accelerated heart rate [65, 66].

Topiramate, an antiepileptic drug with weight loss adverse effects, has been deployed as an anti-obesity medication in combination with phentermine after validation of their long-term safety profiles [67]. Topiramate works centrally by blocking neuronal voltage-dependent sodium channels, boosting GABA activity, antagonizing glutamate receptors and inhibiting carbonic anhydrase, leading to weight loss via lower calorie intake [65, 67, 68].

The combination of phentermine and topiramate in an extended-release pharmacological formulation is officially an FDA-approved medication for the pharmacological treatment of obesity, intended for people with a BMI ≥ 30 kg/m^2^ or with a BMI ≥ 27 kg/m^2^ with obesity-associated comorbidities [69, 70]. Phentermine/topiramate is not approved by the European Medicines Agency (EMA) due to psychiatric and cardiac safety concerns [71].

The maximum dosage of phentermine/topiramate is 15 mg/92 mg, with a 14-day starting dose of 3.75 mg/23 mg, followed by a switch to 7.5 mg/46 mg for 12 weeks. If the weight loss is insufficient, the dose is then increased to 11.25 mg/69 mg for 14 days, followed by a final increase to 15 mg/92 mg. Discontinuation should be made by taking the dose every other day for at least a week before stopping therapy [72].

According to a randomized controlled trial (RCT) meta-analysis, the 56-week weight loss effect of phentermine/topiramate combination was 8.07 kg (95% CI 6.14–9.99), with a weight loss of 8.02 kg (95% CI 6.59–9.45) if the treatment was prolonged to 108 weeks [73]

Phentermine/topiramate has a wide spectrum of adverse effects, including dysgeusia, paresthesia, dry mouth, disturbance in attention, irritability, hypoesthesia, constipation and dizziness, with the odds ratio (OR) of attention disruption being 4.48 (95% CI 2.39–8.41) [74]. Phentermine/topiramate is contraindicated in pregnancy, glaucoma, and hyperthyroidism, and in concomitant use with monoamine oxidase (MAO) inhibitors [69], as well as in patients with severe hepatic impairment, and should be avoided in dialysis patients [75]

### Naltrexone/Bupropion

Both bupropion and naltrexone act by modulating pro-opiomelanocortin (POMC) neurons in the arcuate nucleus of the hypothalamus. Bupropion directly increases POMC production and is therefore a promising target for weight loss treatment in obesity, in which the production of POMC is reduced [76]. Isolated treatment by bupropion downregulates POMC production due to β-endorphin self-inhibitory feedback loop through the μ-opioid receptor (MOP-R) [76]. Naltrexone, a direct central MOP-R antagonist, has been added to the pharmacological formulation to inhibit the feedback loop and potentiate the effect of bupropion [77].

A fixed combination of naltrexone/bupropion is an FDA- and EMA-approved medication for obesity, intended for the treatment of patients with a BMI ≥ 30 kg/m^2^, or a BMI ≥ 27 kg/m^2^ with obesity-associated comorbidities [78]. The maximum daily dose of naltrexone/bupropion is 32 mg naltrexone hydrochloride and 360 mg bupropion, starting from 8 mg/90 mg, with a weekly increase by 8 mg/90 mg up until the maximum tolerated dose [79].

According to a meta-analysis (4 studies, 3952 patients), the weight loss effect of naltrexone/bupropion over 52 weeks of treatment was 5.0 kg (95% CI 4.4–5.5 kg) in excess of placebo [80]. The TWL after using naltrexone/bupropion for 56 weeks bupropion was 7.8% ± 0.4, versus 4.9% ± 0.6 with placebo (*p* < 0.001), according to a randomized trial (*n* = 793, 3:1) [81].

The commonly seen adverse effects of naltrexone/bupropion include nausea (29–42%), constipation (16–24%), headache (14–24%), vomiting (9–18%), dizziness (7–15%), insomnia (8–11%), dry mouth (6–9%) and diarrhea (5–15%), with sporadic severe adverse effects in the form of suicidal thoughts and seizures [82]. Naltrexone/bupropion is also reported to slightly raise blood pressure and heart rate. The use of naltrexone/bupropion is therefore contraindicated in patients with seizure disorder, uncontrolled hypertension, and severe psychiatric disorders, as well as in patients with severe hepatic impairment and end-stage renal failure [82].

### Orlistat

Orlistat is an FDA- and EMA-approved weight-loss medication indicated for patients with a BMI of at least 30 kg/m^2^, or 27 kg/m^2^ in the presence of obesity-associated complications [83, 84]. Orlistat is currently the only weight-loss medication available for over-the-counter purchase and use [85]. No titration of orlistat is necessary and the dose is 120 mg three times daily with major meals [86].

The XENDOS trial (*n* = 3305), a 4-year, double-blind, prospective study of patients with a BMI ≥30 kg/m^2^, has reported the weight loss effect of orlistat after 1 year was 10.6 kg, versus 6.2 kg with placebo (*p* < 0.001), with the weight loss after 4 years being 5.8 kg, versus 3.0 kg with placebo (*p* < 0.001) [87].

Orlistat is a specific inhibitor of pancreatic and intestinal lipases, resulting in the excretion of approximately 30% of ingested lipids, compared with the physiological 5% [88, 89]. The weight loss effect is accomplished both by reducing the dietary energy content and by the passage of undigested lipids through the intestine, leading to steatorrhea and resulting in semi-voluntary exclusion of lipids to avoid diarrhea and stomach ache [90]. A major adverse effect of orlistat is abdominal pain and diarrhea, and also reduced absorption capacity for lipid-soluble vitamins that could potentially lead to their deficiency [90]. Orlistat is contraindicated in patients with cholestasis, chronic malabsorption, and severe renal impairment [91].

### Glucagon-Like Peptide-1 (GLP-1) Receptor Agonists

The newest and most promising approved anti-obesity medications belong to the glucagon-like peptide-1 (GLP-1) agonist class, which are members of a larger incretin mimetics family. GLP-1 agonists were originally developed as an antidiabetic medication, with a weight loss effect being reported during clinical trials [92]. The significant weight loss effect is of special significance in the treatment of type 2 diabetes, in which the addition of some other glucose-lowering agents (insulin and sulphonylurea) commonly led to weight gain and the need for continuous therapy escalation [93]. Current guidelines for the treatment of type 2 diabetes name weight management as one of the core tenets of therapy [94] and GLP-1 receptor agonists (RAs) as the first injectable therapy to be used before the initiation of insulin.

There are multiple mechanisms by which the GLP-1 RAs affect glycemia and body weight. The weight control effect is mainly accomplished by slowing down gastric emptying and by central anorexigenic effect, with both of these effects leading to smaller food portions. The antidiabetic effect is achieved by enhancing glucose-dependent insulin secretion and by inhibiting glucagon secretion [95]. In addition to metabolic effects, GLP-1 RAs also decrease blood pressure and improve dyslipidemia, and for some of them (liraglutide, dulaglutide, semaglutide), cardioprotective and renoprotective properties have been demonstrated in patients with type 2 diabetes. The cardioprotective effects of GLP-1 RAs have been recently validated by the large-scale SELECT (*n* = 17,604; 1:1; 2.4 mg:placebo) semaglutide trial, reporting a significant risk reduction of three-point major cardiovascular events (MACEs)—cardiovascular death, non-fatal myocardial infarction, or non-fatal stroke—by 20% (hazard ratio [HR] 0.80, 95% CI 0.72–0.90; *p* < 0.001) [96].

GLP-1 RAs have also been shown to have potential therapeutic benefit in patients with post-bariatric postprandial hypoglycemia, but at the moment there are limited and incomplete data supporting this indication [97].

At present, two GLP-1 agonists (liraglutide and semaglutide) have been approved by the FDA and EMA for use as an anti-obesity medication regardless of the presence of diabetes [98, 99].

The most common adverse effects associated with GLP-1 agonists are gastrointestinal, with the most common being nausea, and with other adverse effects primarily being headache and nasopharyngitis [100]. The safety profile is similar between semaglutide and liraglutide [101]

GLP-1 agonists are contraindicated in pregnant or breastfeeding women, and in the case of familial or personal history of medullary carcinoma and multiple endocrine neoplasia type 2 (MEN2) syndrome [102]. Special consideration should also apply to patients with heightened pancreatitis risk [103, 104].

#### Liraglutide

After its approval by the FDA as a primary weight loss drug in 2014 for patients with a BMI of at least 30 kg/m^2^, or 27 kg/m^2^ in the presence of obesity-associated comorbidities, liraglutide has become a mainstay medication for weight loss [102]. Liraglutide is available as a once-daily subcutaneous injection and the approved maximum dose of liraglutide is 1.8 mg for diabetes and 3.0 mg for obesity [102]. Titration starts from 0.6 mg, with a weekly increment by 0.6 mg until reaching the maximum tolerated dose [105].

The LEADER trial, a randomized, multicenter, double-blind, placebo-controlled study of liraglutide 1.8 mg in patients (*n* = 9340) with type 2 diabetes mellitus, has reported the weight loss effect of liraglutide 1.8 mg after 36 months was 2.3 kg (95% CI 2.5–2.0) when compared with placebo [106].

The largest randomized, controlled, double-blind trial concerning the use of liraglutide in a 3.0 mg dose (*n* = 3731, 2:1) in people without diabetes achieved a weight loss of 8.4 ± 7.3 kg by the end of week 56, with those in the placebo group losing a mean of 2.8 ± 6.5 kg (a difference of 5.6 kg; 95% CI 6.0–5.1; *p* < 0.001, with last-observation-carried-forward imputation). The TWL was 8.0 ± 6.7% in the liraglutide group, while those in the placebo group had a TWL of 2.6 ± 5.7%, with the estimated treatment difference being 5.4% (95% CI 5.8–5.0; *p* < 0.001, with last-observation-carried-forward imputation) [107]. Other trials have confirmed the weight reduction effect [108–113], including one retrospective trial in a South Korean population (*n* = 169, BMI > 27 kg/m^2^) reporting a weight loss of 7.8 ± 3.5 kg after 180 days (other timepoints: 30 days, 3.2 ± 1.8 kg; 60 days, 4.5 ± 2.3 kg; and 90 days, 6.3 ± 2.6 kg). The TWL after 180 days in this study was 9.1 ± 3.6% (other timepoints: 30 days, 3.8 ± 2.1%; 60 days, 5.3 ± 2.6%; and 90 days, 7.5 ± 2.9%) [114].

#### Semaglutide

The second GLP-1 agonist approved for use as an anti-obesity medication is semaglutide, first approved by the FDA as an antidiabetic drug in 2017 [115], closely followed by other agencies. While originally developed as a once-weekly subcutaneous injection, a once-daily oral formulation was approved by the FDA in 2019 [116]. As was the case with liraglutide, a higher dosage of subcutaneous semaglutide intended for obesity treatment was developed and approved in 2021 [99]. The approved maximum dose of semaglutide is 2.0 mg for diabetes and 2.4 mg for obesity. Dose adjustment of the subcutaneous 2.4 mg formulation is performed monthly, in a sequence of 0.25, 0.5, 1, 1.7 and 2.4 mg, or until reaching the maximum tolerated dose [117].

A 68-week, double-blinded trial (*n* = 1961) in patients with obesity without diabetes (STEP 1) has reported the weight loss effect of semaglutide 2.4 mg was 15.3 kg, compared with 2.6 kg in the placebo group (estimated treatment difference, 12.7 kg; 95% CI 13.7–11.7) [118]. The TWL over 68 months was 14.9% in the semaglutide group, compared with 2.4% when using placebo, for an estimated treatment difference of 12.4% (95% CI 13.4–11.5; *p* < 0.001). However, it should also be noted that the extension of the STEP 1 trial to 120 weeks has reported a significant weight regain of 11.6% (standard deviation [SD] 7.7) of baseline weight 52 weeks after semaglutide withdrawal, confirming the need for long-term continuous treatment even after the initial weight loss [119]

This finding is in agreement with a meta-analysis of 41 randomized controlled trials, comparing the effectiveness of semaglutide 2.4 mg with phentermine/topiramate and placebo [120]. The TWL difference between phentermine/topiramate 92 mg/15 mg and semaglutide 2.4 mg was − 0.40% (credible interval [CrI] − 4.22 to 3.46), with the difference between placebo and semaglutide being − 9.36% (CrI − 12.06 to − 6.62) [120]. STEP 2 and STEP 3 trials have also confirmed a significant weight loss effect [121, 122].

### Tirzepatide

Tirzepatide is a compound belonging to the incretin mimetic family, intended for the treatment of type 2 diabetes and obesity [123]. Tirzepatide first received FDA and EMA approval for use as an antidiabetic medication in 2022, which was followed by FDA approval for the treatment of obesity in 2023 [124]. In contrast to GLP-1 RAs, tirzepatide is a dual agonist of both GLP-1R and glucose-dependent insulinotropic polypeptide-receptor (GIP-R) [125]. Tirzepatide is available as a once-weekly subcutaneous injection, with a maximum dose of 15 mg. Dose adjustment is monthly, with a starting dose of 2.5 mg and a monthly increase of 2.5 mg until reaching the maximum tolerated dose [126].

Tirzepatide has shown both a significant improvement in glycemic control and a marked weight loss effect. A 72-week, phase III, double-blind, randomized controlled trial investigating the use of tirzepatide (SURMOUNT-1) in patients with overweight or obesity (*n* = 2539; 1:1:1:1; 5 mg:10 mg:15 mg:placebo) without diabetes mellitus has been completed. This study has shown a body weight reduction of 16.1 kg and a TWL of 16.0% (95% CI 16.8–15.2) with the 5 mg dose; a reduction of 22.2 kg and a TWL of 21.4% (95% CI 22.2–20.6) with the 10 mg dose; and a reduction of 23.6 kg and a TWL of 22.5% (95% CI 23.3–21.7) with the 15 mg dose; the mean reduction with placebo was 2.4 kg, with a mean TWL of 2.4% (95% CI 3.2–1.6) [125].

A second phase III, double-blind, randomized, controlled, anti-obesity study of tirzepatide (SURMOUNT-2, *n* = 1514; 1:1:1; 10 mg:15 mg:placebo) has confirmed the efficacy of 72 weeks use of tirzepatide in patients with diabetes and either overweight or obesity [127]. SURMOUNT-2 showed a least-squares mean percentage change weight loss at week 72 of 12.9 kg and a TWL of 12.8% (standard error [SE] 0.6) with tirzepatide 10 mg; 14.8 kg and a TWL of 14.7% (SE 0.5) with tirzepatide 15 mg; and 3.2 kg and a TWL of 3.2% (SE 0.5) with placebo. Both tirzepatide doses were superior to placebo, with estimated weight loss treatment differences in comparison with placebo of 9.7 kg (95% CI 11.2–8.2; *p* < 0.0001) for the 10 mg dose and 11.6 kg (95% CI 13.1–10.2; *p* < 0.0001) for the 15 mg dose.

Adverse effects, as well as contraindications, are similar to those of GLP-1 agonists [123]. The rate of adverse events was similar with semaglutide and tirzepatide, with a rate of serious adverse events of 5–7 % in patients receiving tirzepatide and 3% in patients receiving semaglutide [128].

A summary of clinical trials concerning the use of anti-obesity medication in non-bariatric population can be reviewed in Table 2.

**Table 2:** Summary of trials investigating the efficacy of anti-obesity medication in a non-bariatric population

Medication	Maximum approved dose	Trial dose	Weight loss effect	Duration of treatment	Adverse effects—types	Contraindications	Trial type	Sample size, characteristics	References
Phentermine/topiramate	15 mg/92 mg	15 mg/92 mg	8.07 kg (95% CI 6.14–9.99)	56 weeks	Dysgeusia, paresthesia, dry mouth, disturbance in attention, irritability, hypoesthesia, constipation and dizziness	Pregnancy, glaucoma, hyperthyroidism, concomitant use with MAO inhibitors, severe hepatic impairment, should be avoided in dialysis	RCT meta-analysis, placebo-controlled	Not specified	[73]
			8.02 kg (95% CI 6.59–9.45)	108 weeks				Not specified	
Naltrexone/bupropion	32 mg/360 mg	32 mg/360 mg	5.0 kg (95% CI 4.4–5.5 kg)	52 weeks	Nausea, constipation, headache, dizziness, insomnia, dry mouth, diarrhea, sporadic suicidal thoughts and seizures	Seizure disorder, uncontrolled hypertension, severe psychiatric disorders, severe hepatic impairment and end-stage renal failure	RCT meta-analysis, placebo-controlled	3952, people without diabetes	[80]
		32 mg/360 mg	TWL of 7.8 ± 0.4% vs. 4.9 ± 0.6 % with placebo	56 weeks			RCT, placebo-controlled	793 (3:1), people without diabetes	[121]
Orlistat	120 mg three times daily	120 mg three times daily	10.6 kg vs. 6.2 kg with placebo (*p* < 0.001)	1 year	Diarrhea and stomach ache, hypovitaminosis	Cholestasis, chronic malabsorption, severe renal impairment	RCT, placebo-controlled	3305 (1:1), people without diabetes	[87]
			5.8 kg vs. 3.0 kg with placebo	4 years					
Liraglutide	3.0 mg	1.8 mg	2.3 kg (95% CI 2.5–2.0)	36 months	Nausea, headache, and nasopharyngitis	Pregnancy, breastfeeding, familial or personal history of medullary carcinoma and MEN2 syndrome	RCT, placebo-controlled	9340 (1:1), people with diabetes	[106]
		3.0 mg	8.4 ± 7.3 kg, placebo 2.8 ± 6.5 kg (difference of 5.6 kg; 95% CI 6.0–5.1; *p* < 0.001) TWL of 8.0 ± 6.7% in the liraglutide group, placebo 2.6 ± 5.7% (difference of 5.4%; 95% CI 5.8–5.0; *p* < 0.001)	56 weeks			RCT, placebo-controlled	3731 (2:1), people without diabetes	[107]
		1.8 mg	Mean TWL of 4.7% (5.0 kg), placebo 2.0% (2.2 kg)	56 weeks			RCT, placebo-controlled	846 (2:1:1), people with diabetes	[156]
		3.0 mg	Mean TWL of 6.0% (6.4 kg), placebo 2.0% (2.2 kg)	56 weeks			RCT, placebo-controlled	846 (2:1:1), people with diabetes	
		3.0 mg + intensive behavioral therapy	Mean TWL of 7.5%. Estimated TWL treatment difference vs. placebo 3.4% (95% CI 5.3–1.6)	56 weeks			RCT, placebo-controlled	282 (1:1), people with and without diabetes	[121]
		3.0 mg + basal insulin	Mean TWL of −5.8%. Estimated TWL treatment difference vs. placebo 4.3% (95% CI 5.5–3.2)	56 weeks			RCT, placebo-controlled	396, (1:1), people with diabetes	[111]
		3.0 mg	7.8 ± 3.5 kg after 180 days (other timepoints: 30 days: 3.2 ± 1.8 kg; 60 days: 4.5 ± 2.3 kg; 90 days: 6.3 ± 2.6 kg) The TWL after 180 days was 9.1 ± 3.6% (other timepoints: 30 days: 3.8 ± 2.1%; 60 days: 5.3 ± 2.6%; 90 days: 7.5 ± 2.9%)	180 days			Retrospective	169, people with and without diabetes	[114]
		3.0 mg	Mean TWL of −6.1% (SD 7.3). Estimated TWL treatment difference vs. placebo 4.3% (95% CI 4.9–3.7)	160 weeks			RCT, placebo-controlled	2254, (2:1), people with prediabetes	[113]
		3.0 mg	Mean TWL of 6.2% (SD 7.3). Estimated TWL treatment difference vs. placebo 6.1% (95% CI 7.5–4.6)	56 weeks			RCT, placebo-controlled	442, (1:1), people with prediabetes	[112]
Semaglutide	2.4 mg	2.4 mg	15.3 kg, placebo 2.6 kg (difference of 12.7 kg; 95% CI 13.7–11.7)	68 weeks			RCT, placebo-controlled	1961 (2:1), people with diabetes	[118]
		2.4 mg	Mean TWL of 16.0%. Estimated TWL treatment difference vs. placebo 10.3% (95% CI 12.0–8.6)	68 weeks			RCT, placebo-controlled	611 (2:1), people without diabetes	[121]
		1.0 mg and 2.4 mg	Mean TWL of 9.6% (SE 0.4) with 2.4 mg vs. 7.0% (SE 0.4) with 1.0 mg vs. 3.4% (SE 0.4) with placebo. Estimated TWL treatment difference for semaglutide 2.4 mg vs. semaglutide 1.0 mg was 2.7 % (95% CI 3.7–1.6)	68 weeks			RCT, placebo-controlled	1210 (1:1:1), people with diabetes	[122]
		2.4 mg	TWL difference of semaglutide vs. phentermine/topiramate was −0.40% (CrI −4.22 to 3.46), while semaglutide vs. placebo was −9.36% (CrI −12.06 to −6.62)	52 weeks			RCT meta-analysis	41 RCTs, people without diabetes	[120]
Tirzepatide	15 mg	5 mg	16.1 kg, a TWL of 16.0% (95% CI 16.8–15.2)	72 weeks			RCT, placebo-controlled	2539, 1:1:1:1, people without diabetes	[125]
		10 mg	22.2 kg, a TWL of 21.4% (95% CI 22.2–20.6)						
		15 mg	23.6 kg, a TWL of 22.5% (95% CI 23.3–21.7); reduction with placebo was 2.4 kg, with a mean TWL of 2.4% (95% CI 3.2–1.6)						
		10 mg	12.9 kg or 12.8% of TWL (SE 0.6)	72 weeks			RCT, placebo-controlled	1514, 1:1:1, people with diabetes	[127]
		15 mg	14.8 kg or 14.7% (SE 0.5) and 3.2 kg or 3.2% of TWL (SE 0.5) with placebo						

*CI* confidence interval, *CrI* credible interval, *MAO* monoamine oxidase, *MEN2* multiple endocrine neoplasia, *RCT* randomized controlled trial, *SD* standard deviation, *SE* standard error, *TWL* total weight loss

## Use of Anti-obesity Medications as an Augmentation of Bariatric Surgery

### Phentermine/Topiramate

Both phentermine and topiramate have been clinically proven to prevent weight regain after bariatric surgery, either in combination or when used separately. A retrospective analysis [129] (*n* = 760; 350 patients with anti-obesity medication) has shown that the weight regain of patients after RYGB in patients using either phentermine/topiramate (*n* = 154), topiramate (*n* = 74), or phentermine (*n* = 119) has been approximately 10% lower at the end of the 6-year observation period when compared with the control group. No distinction between prescribed drugs was made during the analysis. When considering weight regain on a monthly basis, the HR of weight regain of 1.22% relative to nadir per month was 0.729 (CI 0.556–0.957; *p* = 0.023) compared with the control group [129]. However, that study did not monitor the odds or severity of adverse events in this group of patients.

Another retrospective analysis [157] reported a significant weight loss effect of phentermine/topiramate in patients with weight regain or weight loss plateau after either RYGB or LAGB. The average weight loss at 90 days was 3.8 kg (95 % CI 1.08–6.54; 12.9% EWL) when using phentermine/topiramate. However, that is limited by high variability in both the period between surgery and the time of nadir, and the period between nadir and the beginning of pharmacotherapy [157]. No serious adverse effects were reported.

### Naltrexone/Bupropion

While the Canadian guidelines offer the option of using naltrexone/bupropion for the treatment of obesity in patients with bariatric surgery [130], at present there are no completed randomized controlled trials evaluating the use of naltrexone/bupropion on weight management after bariatric surgery.

One retrospective study (*n* = 209) [131] of patients using either phentermine (*n* = 156), phentermine/topiramate (*n* = 25), lorcaserin (*n* = 18), or naltrexone/bupropion (*n* = 10) in patients after RYGB, SG or LAGB was conducted. While this study has reported an additional weight loss of 2.4 kg over 12 months and TWL of 2.2% after the initiation of therapy, it did not differentiate between the type of medication, of which naltrexone/bupropion represented only 4.8% [131].

One randomized controlled trial is currently undergoing recruitment and is evaluating the effectiveness of naltrexone/bupropion patients who underwent RYGB or SG with 5% weight gain after reaching nadir [132]. No preliminary findings have been reported to date.

### Orlistat

The only trial investigating the effect of orlistat after bariatric surgery was a pair-matched study comparing the use of orlistat with placebo in patients (*n* = 38) 18 ± 6 months after laparoscopic adjustable gastric banding (AGB), who stopped losing weight 3 months before the initiation of the trial therapy [133]. This study has demonstrated an 8-month weight loss of 8 ± 3 kg in the orlistat group versus 3 ± 2 kg in the placebo group (*p* < 0.01), with a TWL of 8.2% in the orlistat group. After extension of the trial by 9 months in patients initially using orlistat, weight remained unchanged in both the placebo and orlistat groups [133]. This small study thus proved that orlistat might be useful in patients after gastric banding, a purely restrictive surgery. On the other hand, its use after malabsorptive surgery has not been tested and it therefore cannot be recommended after this type of operation.

### GLP-1 Agonists

#### Liraglutide

Liraglutide has been extensively studied as an adjuvant medication in patients after bariatric surgery. A randomized, double-blind, placebo-controlled, multicentric trial (GRAVITAS; *n* = 71, 2:1, liraglutide:placebo) [134] studied the effect of liraglutide 1.8 mg in patients with recurrent or persistent type 2 diabetes mellitus 1 year after RYGB. The GRAVITAS trial showed a 4.23 kg (6.81 kg vs. 1.64 kg; *p* = 0.0017) weight loss difference between the liraglutide 1.8 mg and placebo groups after 26 weeks of treatment [134]. No serious adverse effects related to liraglutide treatment were reported, with the incidence of the most common adverse effect, nausea, being similar in the placebo and liraglutide groups (RYGB: 16% vs. 19%; SG: 13% vs. 9%; placebo vs. liraglutide) [134].

A second randomized, double-blinded, controlled trial of patients (*n* = 23, 1:1) [135] following SG explored the use of liraglutide 3.0 mg as an augmentation to bariatric surgery 6 weeks after operation. After 24 weeks of treatment, weight loss was higher in the laparoscopic sleeve gastrectomy [LSG] + liraglutide group, with an EWL of 58.7% ± 14.3, compared with 44.5% ± 8.6 in the LSG-only group (*p* = 0.043). The TWL of the LSG + liraglutide group was 28.2 ± 5.7% (*p* = 0.116), while the TWL of the LSG group was 23.2 ± 6.2%. BMI decreased by 11.7 ± 3.5 kg/m^2^ in the LSG + liraglutide group (*p* = 0.287) compared with 9.5 ± 4.0 kg/m^2^ in the LSG group. Neither difference in BMI or TWL reached statistical significance, very likely due to the small sample size. No statistically significant difference was reported for the incidence of nausea (41.7% and 36.4%; *p* = 0.765) or vomiting (33.3% and 27.3%; *p* = 0.655) between the groups [135].

A randomized, double-blinded, controlled trial of patients with weight regain at least 18 months after RYGB using liraglutide uptitrated to 3.0 mg (*n* = 82, 2:1 liraglutide 3.0 mg vs. placebo) with or without diabetes has been completed but has not yet been published. The preliminary reports have revealed that 67.2% of patients in the liraglutide arm lost at least 5% of body weight after 12 months of treatment, versus 4.4% in the placebo arm [136], with no significant difference in adverse effects between the groups.

The conclusions of randomized trials have been complemented by numerous retrospective studies. A retrospective trial (*n* = 117) in patients using liraglutide 3.0 mg for up to 8 years (7.8 ± 5.7 years, IQR 4–10 years) after bariatric surgery (RYGB, SG or LAGB) has shown a mean body weight loss of 5.5 ± 6.2% (6.3 ± 7.7 kg; *p* < 0.05) over a period of 7.6 ± 7.1 months [137]. A second retrospective trial that included patients both without (*n* = 711) and after bariatric surgery (*n* = 76) using liraglutide 3.0 mg for ≥16 weeks reported that the median (IQR) weight loss was 6.0 kg (2.4–9.4), equivalent to 6.4% (2.6–9.7%) of baseline weight (*p* < 0.0001, *n* = 787), with no significant difference in weight loss between post-bariatric and non-bariatric patients [138]. Another retrospective study (*n* = 62) has shown a significant weight loss after 10.5 ± 4.4 months of liraglutide 3.0 mg treatment in patients with weight regain after RYGB, LSG, and LAGB, with a mean BMI loss of 5.1 ± 2.5 kg/m^2^ [139].

A retrospective study (presented as a poster) [*n* = 49] of patients after bariatric surgery (RYGB, SG, or LAGB) reported a short-term weight loss of 5.4 ± 9.8 kg in patients undergoing 3 months of liraglutide 3.0 mg therapy [140]. Other available studies have shown both the efficacy and safety of the use of liraglutide in patients after bariatric surgery [141–143]

In addition, a retrospective study [144] has confirmed the effectiveness and safety of liraglutide 3.0 mg in patients after a secondary bariatric operation, with no significant difference between the primary and secondary patient groups. Body weight loss after 12 months of liraglutide 3.0 mg was 6.50 ± 7.58 kg for primary patients and 4.90 ± 8.91 kg for secondary patients, with no statistically significant difference between both groups of patients (*p* = 0.35) [144].

#### Semaglutide

While no randomized, placebo-controlled trial of semaglutide in patients after bariatric surgery has been completed and published, retrospective studies have confirmed a significant weight loss effect of semaglutide in patients with weight regain following bariatric surgery.

One retrospective trial has investigated the effect of semaglutide 0.5 mg treatment in patients with weight regain or insufficient weight loss 64.7 ± 47.6 months after bariatric surgery (RYGB or SG). TWL after 3 months (3.2 months, IQR 3.0–3.5, *n* = 38) of treatment was 6.0 ± 4.3 % (mean ± SD; *p* < 0.001), with TWL of 10.3 ± 5.5% (mean ± SD; *p* < 0.001) after 6 months (5.8 months, IQR 5.8–6.4, *n* = 20) [145].

A retrospective observational study comparing the use of semaglutide and liraglutide for 6 months in patients 72.0 months (43.8–96.0) after bariatric surgery (RYGB, SG, gastric banding) has been published, demonstrating a higher effect of semaglutide compared with liraglutide, even in its 1.0 mg subcutaneous (or 14 mg oral) dose when compared with a 3.0 mg dose of liraglutide, with a BMI reduction of 3.9 kg/m^2^ (2.9–4.8) versus 2.5 kg/m^2^ (1.1–3.3) [semaglutide vs. liraglutide; *p* < 0.001]. The reported TWL was 9.8% (8.2–13.0) with semaglutide and 7.3% (3.1–10.3) with liraglutide (*p* < 0.05) [146].

Similar results were reported by another retrospective study of post-bariatric patients (RYGB, SG, gastric banding) using either liraglutide 3.0 mg or semaglutide 1.0 mg subcutaneously for 12 months, with a reported least-squares mean weight loss at 12 months of 12.92% and 8.77% in the semaglutide and liraglutide groups, respectively (*p* < 0.001) [147].

### Tirzepatide

While tirzepatide has recently been approved [124] as an anti-obesity medication in light of the positive outcomes of the SURMOUNT-1 and SURMOUNT-2 studies [125, 127, 148], at present no retrospective or prospective trials have studied the use of tirzepatide in patients after bariatric surgery.

A summary of clinical trials concerning the use of anti-obesity medication in post-bariatric population can be reviewed in Table 3.

**Table 3:** Summary of trials investigating the efficacy of anti-obesity medication in a post-bariatric population

Medication	Maximum approved dose	Trial dose	Weight loss effect	Duration of treatment	Surgery type	Guidelines	Trial type	Sample size, characteristics	References
Phentermine/topiramate	15 mg/92 mg	Unknown	All anti-obesity medication users – weight regain 10% lower at the end of the 6-year observation period when compared with the control group when using nadir as a baseline	6 years	RYGB	AACE	Retrospective	760 [350 with anti-obesity medication; phentermine/topiramate (*n* = 154), topiramate (*n* = 74), or phentermine (*n* = 119)]	[129]
		7.5 mg/46 mg	3.8 kg (12.9 % EWL; 95% CI 1.08–6.54)	90 days	RYGB + LAGB		Retrospective	13, people with and without diabetes	[157]
Naltrexone/bupropion	32 mg/360 mg	32 mg/360 mg	−2.4 kg, a TWL of 2.2%	12 months	RYGB + SG + LAGB	Canadian guidelines	Retrospective	209 (10 using naltrexone/bupropion), people with and without diabetes	[131]
Orlistat	120 mg three times daily	120 mg three times daily	8 ± 3 kg in the orlistat group vs. 3 ± 2 kg in the placebo group (*p* < 0.01). The TWL in the orlistat group is 8.2%	8 months, if extended by 9 months no significant change	LAGB	Canadian guidelines – warning against use after malabsorptive surgery	RCT, pair-matched, placebo-controlled	38 (1:1), diabetes status unspecified	[133]
Liraglutide	3.0 mg	1.8 mg	4.23 kg (6.81, 1.64; *p* = 0.0017) compared with placebo	26 weeks	RYGB	AACE, Canadian guidelines	RCT, placebo-controlled	71 (2:1), people with diabetes	[134]
		3.0 mg	Mean EWL of 58.7% ± 14.3, compared with 44.5% ± 8.6 in the LSG-only group (*p* = 0.043)	6 weeks after the surgery, duration 24 weeks	LSG		RCT, placebo-controlled	23 (1:1), people with and without diabetes	[135]
		3.0 mg	67.2% of patients in the liraglutide arm have lost at least 5% vs. 4.4% in the placebo arm	12 months	RYGB		RCT, placebo-controlled, not yet published	82 (2:1), diabetes status unspecified	[136]
		3.0 mg	TWL 5.5 ± 6.2% (6.3 ± 7.7 kg, *p* < 0.05)	7.6 ± 7.1 months	RYGB + SG + LAGB		Retrospective	117, diabetes status unspecified	[137]
		3.0 mg	6 kg (6.4% of baseline body weight), no difference between bariatric and non-surgical patients	30 weeks	Not Specified		Retrospective	Conservative (*n* = 711) and after bariatric surgery (*n* = 76), people without diabetes	[138]
		3.0 mg	Mean BMI change of 5.1 ± 2.5 kg/m^2^	10.5 ± 4.4 months	RYGB + SG + LAGB		Retrospective	62, people without diabetes	[139]
		3.0 mg	5.4 ± 9.8 kg	3 months	RYGB + SG + LAGB		Retrospective	49, diabetes status unspecified	[140]
		3.0 mg	6.50 ± 7.58 kg for primary patients and 4.90 ± 8.91 for secondary patients (*p* = 0.35)	12 months	Primary—LSG, LAGB, RYGB; Secondary—SADI, RYGB, LSG		Retrospective	145 (119/82% primary surgery; 26/18% secondary surgery), people with and without diabetes	[144]
		Maximum tolerated dose	3.26 kg in favor of the liraglutide group after 4 months of treatment (19.23 ± 3.33 kg vs. 22.28 ± 3.26 kg (*p* = 0.002), respectively). After 7 months, weight loss was 20.95 ± 3.21 kg with endoscopy vs. 25.02 ± 3.80 kg (*p* < 0.001) with endoscopy + liraglutide	5 months after surgery, duration of 4 and 7 months	Endoscopic sleeve gastroplasty		Retrospective, pair-matched	52 (1:1), people without diabetes	[158]
Semaglutide	2.4 mg	0.5 mg	TWL of 6.0 ± 4.3%	3 months	RYGB + SG	Only retrospective data, no obvious contraindication	Retrospective	38, people without diabetes	[145]
		0.5 mg	TWL of 10.3 ± 5.5%	6 months	RYGB + SG		Retrospective	20, people without diabetes	
		0.5 mg	BMI loss of 3.9 kg/m^2^ (2.9, 4.8) vs. 2.5 kg/m^2^ (1.1, 3.3), semaglutide 0.5 mg vs. liraglutide 3 mg (*p* < 0.001) TWL of 9.8% (8.2, 13.0) vs. 7.3% (3.1, 10.3), semaglutide 0.5 mg vs. liraglutide 3 mg (*p* < 0.05)	6 months	RYGB + SG + LAGB		Retrospective	50, diabetic patients with and without diabetes	[146]
		1.0 mg	TWL of 12.92% with semaglutide 1.0 mg vs. 8.77% with liraglutide 3.0 mg	12 months	RYGB + SG + LAGB		Retrospective	207, people with and without diabetes	[147]
Tirzepatide	15 mg					Lack of data in post-bariatric patients			

*AACE* American Association for Clinical Endocrinology, *BMI* body mass index, *CI* confidence interval, *EWL* excess weight loss, *LAGB* laparoscopic adjustable gastric banding, *LSG* laparoscopic sleeve gastrectomy, *RCT* randomized controlled trial, *RYGB* Roux-en-Y gastric bypass, *SADI* single anastomosis duodeno-ileostomy, *SE* standard error, *SG* sleeve gastrectomy, *TWL* total weight loss

### The Use of Anti-obesity Medication After Bariatric Surgery According to Current Guidelines

The official 2016 guidelines of the American Association for Clinical Endocrinology (AACE) for the treatment of obesity name phentermine/topiramate and liraglutide as a possible therapy of choice for the attenuation of weight regain after bariatric surgery (Grade D; BEL 3; downgraded due to evidence gaps) [149].

While the 2020 Canadian Adult Obesity Clinical Practice Guidelines do not have a clear recommendation for the use of anti-obesity medication after bariatric surgery, they do mention the possibility of the use of liraglutide and naltrexone/bupropion as adjuvant therapy after bariatric surgery, as demonstrated by retrospective analyses. These guidelines [130] caution against the use of orlistat after malabsorptive surgery, seemingly due to concerns of higher risk of adverse effects (mainly lipid-soluble vitamin deficiency) and redundancy [150, 151].

European Association for Study of Obesity (EASO) guidelines [152, 153] do not present a clear stance on the use of any anti-obesity medication after bariatric surgery.

While retrospective studies support the efficacy of semaglutide treatment in patients after bariatric surgery, no guidelines currently mention the use of semaglutide. Although there is a lack of data from completed randomized controlled trials, data from both the extensive liraglutide trials and retrospective semaglutide studies at least partially prove the possibility of effective and safe use of semaglutide in the post-bariatric group of patients, albeit with the need to exercise increased caution in the absence of more concrete evidence.

Due to the lack of any specific trials concerning the use of tirzepatide in patients after bariatric surgery, and due to the partially different mechanisms of action of GLP-1 RAs and the dual agonist tirzepatide (GLP-1R and GIP-R), no accurate prediction on the safety and efficacy of tirzepatide in patients after bariatric surgery can currently be made. Nevertheless, as is the case with semaglutide, the available evidence does at least partially prove the possibility of effective and safe use of tirzepatide after bariatric surgery.

## Conclusion

Bariatric surgery is the most effective modality of treatment for both obesity and its associated comorbidities, in particular type 2 diabetes mellitus. Anti-obesity medications have an established place in the obesity treatment algorithm, both as a second-line therapy after lifestyle modifications and as neoadjuvant therapy before bariatric surgery, to achieve the necessary pre-bariatric weight loss.

Even in light of the high effectiveness of bariatric surgery, the surgery itself still has some limitations. Weight regain after bariatric surgery or insufficient weight loss after the operation occurs in all types of bariatric surgery, being more frequent in restrictive bariatric surgeries. While in some patients consequent bariatric surgery can be performed, in others another surgery may be deemed too risky. With the advent of new and effective anti-obesity medications, a novel avenue has opened in using weight loss medication as a middle-line solution in a large spectrum of patients suffering from weight regain or insufficient weight loss, and could be used to postpone or even entirely avoid the need for a second bariatric operation. While adjuvant anti-obesity medication is, at present, less efficient than bariatric conversion at remediating weight regain or insufficient weight loss, some data point to a possibility of effective use of anti-obesity medication, especially in patients with insufficient weight loss rather than weight regain after bariatric surgery [154].

With the wide spectrum of anti-obesity medication currently available, great care should be taken to select the correct and individualized medication for each patient, based on the medication’s effectiveness, contraindications, adverse effects, and, ultimately, cost.

While the use of orlistat is to be avoided in patients following malabsorptive surgery, other commonly used anti-obesity medications (phentermine/topiramate, bupropion/naltrexone, liraglutide, semaglutide, tirzepatide) have no general contraindication for use in patients after bariatric surgery.

Phentermine/topiramate, liraglutide and semaglutide have shown a clear benefit in post-bariatric patients, with a statistically significant reduction of weight in patients after all types of surgeries. Tirzepatide has not yet been studied in the post-bariatric population but its effectiveness is of great interest in this group of patients.

Despite relatively limited published data, it can be concluded that anti-obesity medication can serve as an effective adjunct therapy to bariatric surgery to help boost post-bariatric weight loss or prevent weight regain. However, it is important to note that cessation of the anti-obesity medication in patients without bariatric surgery leads to a significant weight regain, and the same outcome can be expected in patients after bariatric surgery.
